# Exacerbated expansive vascular remodeling due to disturbed flow in male mice overexpressing protein disulfide isomerase‐A1

**DOI:** 10.14814/phy2.71088

**Published:** 2026-08-30

**Authors:** Júlia Martins Felipe de Souza, Tiphany Coralie De Bessa, Amanda de Almeida Silva, Carolina Gonçalves Fernandes, Leonardo Yuji Tanaka, Francisco Rafael Martins Laurindo

**Affiliations:** ^1^ Vascular Biology Lab (LVascBio), LIM‐64, Heart Institute University of São Paulo School of Medicine (FMUSP) São Paulo São Paulo Brazil; ^2^ Laboratory of Hypertension, Heart Institute (InCor) University of São Paulo School of Medicine São Paulo São Paulo Brazil; ^3^ Santa Casa de São Paulo, School of Medical Sciences (FCMSCSP) São Paulo São Paulo Brazil; ^4^ Redox Mechanobiology Laboratory Federal University of São Paulo (UNIFESP), School of Medicine São Paulo São Paulo Brazil

**Keywords:** extracellular matrix, protein disulfide isomerase A‐1, vascular remodeling

## Abstract

Vascular expansive remodeling is involved in atherosclerosis/restenosis; however, its mechanisms need further investigation. Protein‐disulfide isomerase (PDIA1), a redox chaperone from the endoplasmic reticulum and cell surface, supports vascular smooth muscle mechanoadaptation. Vascular injury repair reportedly associates with marked PDIA1 overexpression, while PDIA1 inhibition limits expansive remodeling. However, the effects of overexpressed PDIA1 in vessel remodeling are unknown. Here, we investigate the effects of transgenic constitutive PDIA1 overexpression (TgPDIA1) in carotid artery expansive remodeling in mice subjected to partial left carotid artery ligation. The ensuing disturbed flow was documented by ultrasound imaging. TgPDIA1 mice exhibited significantly larger total calibers 14 days after ligation, along with increased medial, neointimal, and total wall areas, compared with wild‐type mice. These changes were exacerbated in proximal carotid, coincident with more disturbed flow. ECM analysis revealed reduced birefringent collagen in TgPDIA1 arteries, despite increased total collagen content, indicating enhanced amount but impaired collagen organization. Morphometry disclosed enhanced collagen fiber curvature and reduced elastin fiber thickness in TgPDIA1 ligated arteries. Immunohistochemistry depicted reduced α‐smooth muscle actin and increased vimentin in TgPDIA1 vessels. Analogous expansive remodeling occurred in another series of mice 28 days after total carotid occlusion. These data corroborate the PDIA1 role as a mediator of expansive vascular remodeling.

## 1 INTRODUCTION

Vascular remodeling comprises multiple physiological or disease‐related adaptations and, in conduit vessels, merges with *strictu* sensu remodeling characterized by changes in whole vessel caliber, commonly evaluated as the area enclosed by the external elastic lamina (Pasterkamp et al., 2000). Expansive (outward) remodeling may occur in the complete absence of plaques, for example, due to increased laminar shear stress (Chatzizisis et al., 2007; Deng et al., 2025; Langille & O'Donnell, 1986) while also as a clinically useful marker of complicated atheromas (Davies, 1995; Libby, 2021) and during atherogenesis, in which vessel expansion can buffer lumen encroaching by the plaque (Glagov et al., 1987). Constrictive (inward) remodeling, in turn, occurs during arterial injury repair and is the main mechanism of restenosis after angioplasty (Ward et al., 2000). The pathways underlying such *strictu* sensu remodeling, however, are not well established. Redox processes have emerged as crucial mechanisms of vascular disease and, in particular, of vascular remodeling, as we proposed (Tanaka & Laurindo, 2017). For instance, we showed previously that superoxide dismutase underactivity decreases nitric oxide bioactivity and supports constrictive remodeling in the late vascular repair reaction (Leite et al., 2003). However, cellular integrative mechanisms of such redox communication remain unclear. The endoplasmic reticulum (ER) is an important redox‐active organelle able to connect with other subcellular locations and extracellular milieu, via ER oxidoreductases such as Ero1alpha and the Protein Disulfide Isomerases (PDIs) (Oliveira et al., 2026). We have worked with the hypothesis that PDIs are upstream regulators of vascular remodeling. PDIs are redox chaperones characterized by thioredoxin‐fold motifs. The family contains ca.24 members and its prototype is PDIA1 (Okumura et al., 2015; Wang et al., 2012; Wilkinson & Gilbert, 2004). We showed previously that PDIA1 is required as regulator of several processes known to play roles in vascular remodeling, such as growth factor‐dependent activation of Nox NADPH oxidases (Janiszewski et al., 2005), RhoGTPase activation (Pescatore et al., 2012) and VSMC migration (Tanaka et al., 2019). PDIA1 expression correlates with vulnerable atheromas in humans, which often exhibit expansive remodeling (Muller et al., 2013). The extracellular (cell surface and secreted) pool of PDIA1 regulates VSMC mechanoadaptation and persistence of directional migration (Tanaka et al., 2019). Moreover, extracellular PDIA1 neutralization in vivo with a perivascular antibody promotes constrictive remodeling in the late stages of vascular response to overdistension injury, indicating that PDIA1 supports an expanded vessel structure in this situation, in line with its effects in collagen organization (Tanaka et al., 2016). Importantly, PDIA1 is strongly overexpressed (25‐fold average increase) after vascular injury, particularly during the resolving stage (Tanaka et al., 2016). Furthermore, sustained inducible PDIA1 overexpression in cultured VSMC promotes shifts in Nox subtype expression and VSMC phenotype markers (Fernandes et al., 2020). However, the specific effects of PDIA1 overexpression in vessel remodeling are unclear. Here, we further advance into this question by investigating the effects of forced genetic overexpression of PDIA1 on vascular remodeling. We use a characterized mouse model of global transgenic PDIA1 overexpressionl (Fernandes et al., 2020; Tanaka et al., 2026) in which vessel remodeling is promoted by partial carotid artery ligation (Nam et al., 2009) in normolipidemic mice. Our results provide evidence that PDIA1 overexpression exacerbates expansive vascular remodeling, further reinforcing a general role for PDIA1 as a mechanism per se sufficient to support an open vessel structure.

## 2 METHODS

### 2.1 Mouse model of partial carotid artery ligation

#### 2.1.1 Ethics statement

This study was conducted in accordance with established guidelines of the Conselho Nacional de Controle de Experimentação Animal (CONCEA, Ministry of Science and Technology of Brazil) and was approved by an Ethics Committee of the University of São Paulo School of Medicine (CEUA n° 1661/2021).

#### 2.1.2 Animal studies

**FIGURE 1:**
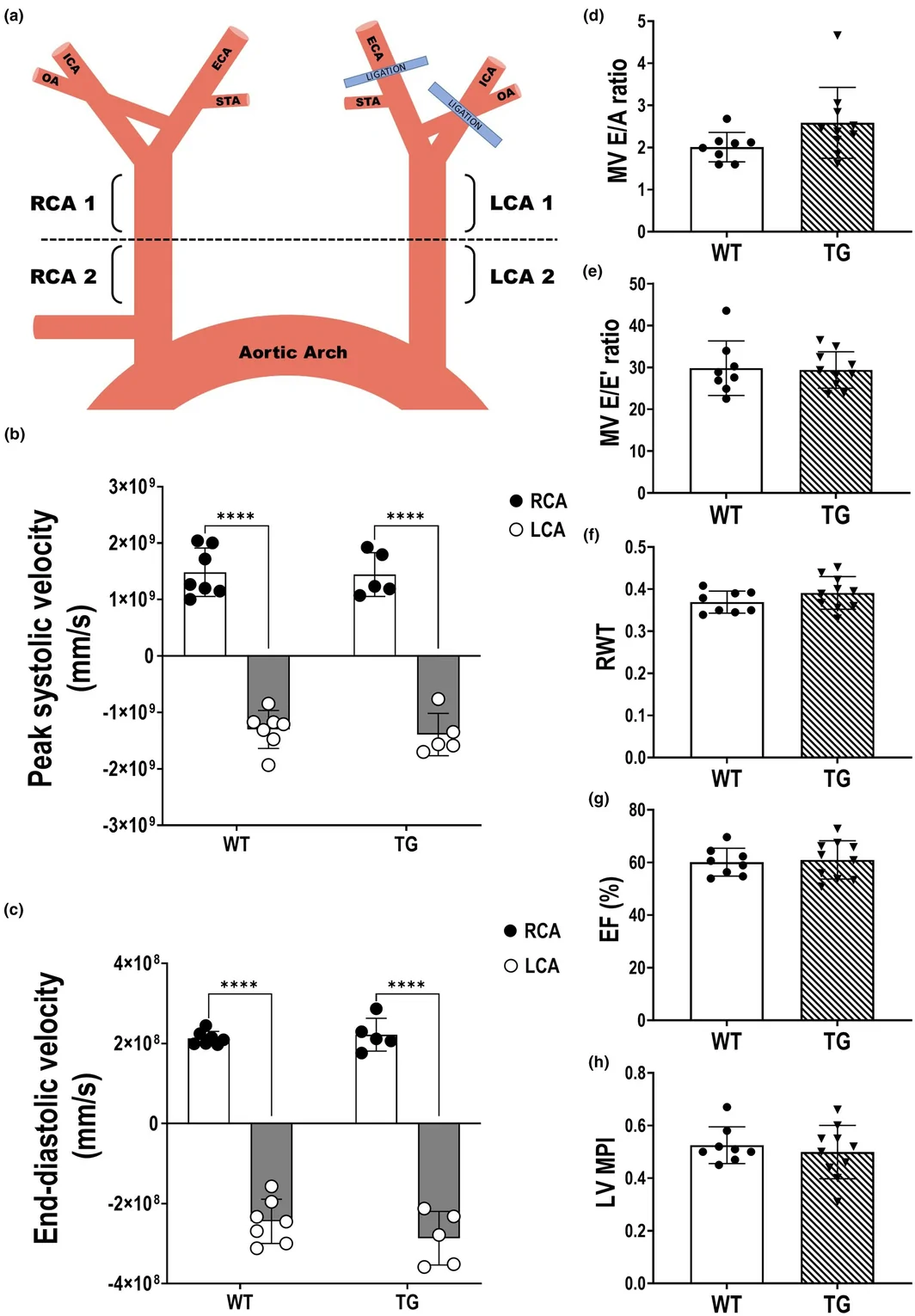
Characterization of Partial Left Carotid Ligation (PCL) model. (a) Schematic representation of the PCL model with the tied branches and the carotid artery segmentation into proximal to the heart (LCA2) and distal (LCA1) regions used for histomorphometric analyses, as well as RCA2 and RCA1. Adapted from (Nam et al., 2009); (b, c) Quantification of (b) peak systolic and (c) end‐diastolic blood flow velocity in right (RCA) and left (LCA) carotid artery demonstrating a marked reduction after PCL, with negative peak values in the LCA, consistent with oscillatory/bidirectional flow. No significant differences were detected between WT and TgPDIA1 mice; (d–h) Echocardiographic parameters demonstrating similar cardiac structure and function in TG mice compared with WT controls. No significant differences were observed between groups for (d) mitral valve E/A ratio, (e) mitral valve E/E′ ratio, (f) relative wall thickness (RWT), (g) ejection fraction (EF), or (h) left ventricular mass index (LVMI). WT *N* = 8; TG *N* = 10. **** *p* < 0.0001.

Mice were housed in a temperature‐ and humidity‐controlled animal facility in individually ventilated cages (Alesco, São Paulo, Brasil; #000079) with red mice shelter for environmental enrichment (Alesco, São Paulo, Brasil; #000155), mounted on ventilated racks (Alesco #144), in a 12 h light/dark cycle with standard chow diet (Nuvilab CR‐1 from Quimtia, Paraná, Brasil; #100110075). Animal studies were performed in mice from a genetic FVB background with global constitutive overexpression of PDIA1 (TgPDIA1) (Fernandes et al., 2020) or their wild type (WT) counterparts. To prevent increased number of experimental groups and considering some temporary limitations of our animal facility, only male mice were used in this study, while females were used for another work in progress in our group. Sixteen‐week‐old mice, WT or TgPDIA1, were anesthetized first in an induction chamber with 3%–4% isoflurane, subsequently maintained via nose cone at 1.5% isoflurane with supplemental oxygen using a precision vaporizer (Harvard Apparatus, Holliston, MA, USA). Mice were kept warm during the procedure using a heating pad and received buprenorphine (0.1 mg/kg) subcutaneously to alleviate pain for 72 h, with the dose repeated if deemed necessary. The shaved area was cleaned with isopropyl alcohol and povidone‐iodine. The procedures were similar to those previously described in the literature (Nam et al., 2009). A ventral incision of 5 mm was made in the neck to expose left carotid artery branches. The left external carotid, internal carotid and occipital artery were ligated using silk 6–0 suture, preserving the superior thyroid artery (Figure 1a). After surgery, mice were placed in a clean box under a heat lamp and monitored until they regained consciousness and presented normal behavior. Food and water were made readily accessible in the box. During two or fourteen days until sacrifice, mice were daily observed for signs of pain, distress, or postoperative complications. Mice were euthanized by exsanguination under deep anesthesia induced by an intraperitoneal injection of xylazine (10 mg/kg) and ketamine (80 mg/kg). After loss of podal reflexes, blood was collected by right ventricular puncture.

### 2.2 Mouse model of total carotid artery ligation

Twelve to sixteen‐week‐old male mice of genetic FVB background (Fernandes et al., 2020) were anesthetized by intraperitoneal injection of xylazine (10 mg/kg) and ketamine (80 mg/kg) followed by 1.5% isoflurane with supplemental oxygen. Carotid occlusion protocol was followed as described (Kumar & Lindner, 1997). A ventral incision in the neck was made to expose the left common carotid artery and branches. Occlusion was made with a 7–0 prolene suture line next to the carotid bifurcation. Incision was closed and mice were monitored while recovering from surgery. Mice received postoperative care as described above and were euthanized as described above 4 weeks after carotid occlusion.

### 2.3 Doppler ultrasound and echocardiography analysis

Mice were anesthetized with 1.5% inhaled isoflurane with supplemental oxygen and assessed at baseline and again 14 days following carotid ligation. Ultrasound evaluations were conducted using a high‐frequency ultrasound system (VEVO 2100, Visual Sonics, Canada) with a 35 MHz probe, provided by Rede Premium network of the University of São Paulo School of Medicine (FMUSP). Image capture and quantitative analyses adhered to the recommendations of the American Society of Echocardiography Standardization Committee (Mitchell et al., 2019; Zacchigna et al., 2021). Cardiac parameters were measured during diastole and systole in three cardiac cycles. Arterial internal diameters were measured in B‐mode longitudinal images during systole, while pulsed‐wave Doppler ultrasound was used to assess flow velocity at distinct portions of the carotid artery during end‐diastole and peak systole (Anea et al., 2010; Korshunov et al., 2017). All measurements were performed in a blinded fashion.

### 2.4 Histopathology of carotid arteries

Both carotid arteries were examined with emphasis on morphometric parameters, collagen and elastin deposition. Following in situ perfusion for 3 min under physiological pressure with PBS and 4% buffered formalin, the vessels were dissected, cleansed from adventitial tissue (but keeping the adventitia layer), fixed for 24 h in the same solution, embedded in paraffin, and sectioned into 3‐μm slices (three sections per slide). Picrosirius staining was applied to highlight collagen, while Verhoeff–Van Gieson staining was used for elastic fibers. Carotid arteries were divided in segments as shown in Figure 1a. For each mouse, four samples were generated (RCA1, RCA2, LCA1, LCA2). Data were shown for each segment individually or pooled segments (RCA or LCA). Proximal segments (designated as #2) were defined as those closer to the aorta (thus further from the ligated branches).

### 2.5 Morphometric analysis

Verhoeff‐Van Gieson and Hematoxylin–eosin images were obtained in a brightfield microscopy (Leica DM 2500) using a 10× objective. Samples were analyzed as duplicates (two sections of each slide) and analyzed using Fiji (ImageJ) software (Schindelin et al., 2012). The morphometric parameters for partial and total carotid ligation included total caliber (= vessel area, i.e. area enclosed by the external elastic lamina), medial area (calculated as external elastic lamina area minus internal elastic lamina area), lumen area, neointimal area, wall area (calculated as neointimal+medial area), intimal/medial ratio (neointimal/medial area), wall tension index (total caliber/wall area) and wall thickness (distance across the vessel wall). Samples with oblique sections or staining artifacts were excluded from the analysis. All sample quality and morphometric analyses (including the confocal analysis described below) were performed in a blinded fashion.

### 2.6 Cell density

Histological sections (duplicates) stained with hematoxylin and eosin were imaged using a brightfield microscope (Leica DM 2500) at 20× magnification. For each sample, two images were captured. Digital images were analyzed using ImageJ/Fiji (NIH). Regions of interest corresponding to the vessel wall were manually defined, excluding the lumen and surrounding tissue. Color deconvolution (H&E vector) was applied to isolate the hematoxylin signal, and the hematoxylin channel was converted to 8‐bit grayscale. Images were segmented for nuclei identification. Cell density (cells/mm^2^) was calculated as the number of nuclei divided by the analyzed tissue area. Only samples in well‐oriented transverse sections were used to avoid biased results.

### 2.7 Collagen analysis under polarized light

Images from picrosirius–stained sections (Direct Red 80, Sigma‐Aldrich; #365548) were obtained under polarized light using standardized acquisition settings (Junqueira et al., 1979) in a 40× objective (Zeiss AXIOSKOP 2 Plus). Briefly, to optimize signal detection, images (duplicates) of the medial layer were acquired with higher light exposure than those of the adventitial layer, while maintaining identical settings within each layer. For each duplicate, four non‐overlapping fields were captured for the medial or adventitial layer. Because collagen detection under polarized light is very sensitive, samples with staining variations were excluded from analysis. Birefringence properties from the anisotropic organization of collagen fibers result in light refraction under polarized microscopy according to fiber orientation (Bromage et al., 2003). Highly birefringent fibers are densely packed and result in light retardation, appearing in red; low birefringent fibers are loosely packed and appear in green (Spiesz et al., 2011). Highly birefringent (red) and low birefringent (green) collagen fibers were quantified in Fiji/ImageJ by splitting RGB images into red and green channels, respectively. Collagen segmentation was performed using machine learning by Trainable Weka Segmentation plugin. Collagen content was expressed as area fraction (% area), calculated as the collagen‐positive area normalized to the total area of the selected region of interest (ROI).

### 2.8 Collagen and elastin analysis under confocal microscopy

Picrosirius‐stained slides were also analyzed by confocal microscopy. Samples with staining variations were excluded from analysis due to image capture sensitivity. Collagen fibers were detected using a 546 nm laser (red channel) and analyzed in parallel with elastic fiber autofluorescence acquired at 488 nm (green channel) (Dolber & Spach, 1993; Wegner et al., 2017). Images were obtained under water immersion using a 40× objective on an inverted spinning disk confocal microscope (Zeiss Cell Observer SD). For each sample, depending on artery size, between one and four images were needed in order to capture the whole vessel area. For quantitative analysis, all images were standardized and processed using Fiji/ImageJ. RGB images were split; the red channel was used for collagen and the green channel for elastin quantification. Images were segmented using the thresholding method (Moments) applied to the red or green channel. Collagen or elastin content was expressed as a percentage of area occupied by the positive signal. Two regions of interest were analyzed: one comprising the entire vessel wall including part of adventitia, and another restricted to the medial layer only. For each vessel, measurements were averaged to obtain a single representative value per animal.

### 2.9 Collagen fiber morphometry

Individual collagen fibers from confocal images were extracted using the CT‐FIRE software (Bredfeldt et al., 2014). Samples with variation in staining and presenting fibers that were not easily identified in the picture were not analyzed, as fiber analysis requires well‐defined images. Because of the high amount of fibers in the artery, only one image was used as representative per sample. Fiber width and straightness were the variables quantified. Because these parameters are intrinsic geometric descriptors and independent of image orientation, angular normalization was not required. To avoid pseudoreplication due to unequal number of fibers per sample, fibers were used only to calculate summary statistics at the animal level. For each animal and arterial segment (RCA or LCA), the median value of fiber width or straightness was calculated and used as the unit of analysis.

### 2.10 Elastin fiber morphometry

Verhoeff‐Van Gieson images in 10× magnification from morphometry analysis were preprocessed in ImageJ/Fiji for standardization prior to CT‐FIRE software analysis. Slides with variations in staining or sectioning were excluded, as the software requires well‐defined images. Following the same criteria in collagen fiber analysis, only one image (showing total vessel area) was representing each sample. Elastin fibers are stained in black, so the chosen images were converted to 8‐bit, inverted (so black staining turns into white, allowing identification by the software), and subjected to a fixed intensity threshold (190–255), then saved as TIFF files. Elastin fiber width and straightness were quantified using CT‐FIRE. As these metrics are intrinsic geometric descriptors and independent of image orientation, angular normalization was not applied. To avoid pseudoreplication, fiber‐level data were summarized using the percentile P25 value per animal and arterial segment (RCA or LCA), which was used as the unit of analysis.

### 2.11 Immunohistochemistry

Paraffin‐embedded tissue blocks were sectioned at 3‐μm thickness and mounted on glass slides (three sections per slide) for immunohistochemical analysis. Sodium citrate buffer was used for heat‐induced epitope retrieval. Hematoxylin was used or not as counterstaining. Immunostaining was performed using the diaminobenzidine (DAB) chromogenic detection method (Envision Flex Agilent #K800221‐2). The following primary antibodies and dilutions were used in this work: PDI monoclonal (RL90) mouse antibody (Invitrogen #MA3‐019; dilution 1:100); PDI polyclonal (N1N3) rabbit antibody (GeneTex #GTX101468, dilution 1:300); Contractile phenotype marker alpha‐actin polyclonal rabbit antibody (Abcam #Ab5694; dilution 1:100) and mesenchymal phenotype marker vimentin polyclonal rabbit antibody (Abcam #137321; dilution 1:500). Secondary antibodies used in this work were: Dako Omnis EnVision FLEX (#GV823); Rabbit LINKER (#GV809) or Mouse LINKER (#GV821) by Agilent (California, US). Images (duplicates) were obtained in brightfield microscopy (Leica DM 2500) using a 40× objective. For each duplicate, four non‐overlapping fields were captured. Samples with staining variations were excluded from analysis. Images were analyzed by Fiji (ImageJ) using Color Deconvolution plug‐in to isolate DAB staining. For each antibody, a specific segmentation threshold technique was used and standardized to avoid analysis bias.

### 2.12 Statistical analysis

All analyses were carried out in GraphPad Prism version 10.0 (GraphPad Software Inc., La Jolla, CA, USA). Differences were considered significant at *p* < 0.05. Samples group is shown in Table S1. For flow analysis, morphometry, collagen quantification and immunohistochemistry results, data are presented as mean ± standard deviation. Normality test (Shapiro–Wilk) was used to evaluate Gaussian (normal) distribution. Statistical comparisons between TgPDIA1 and WT groups were conducted using unpaired *t‐*test or, when specified, two‐way ANOVA with Tukey's post hoc correction, at 5% significance level. For collagen and elastin fiber analysis, statistical comparisons were performed using planned pairwise comparisons between genotypes within the same arterial segment and between arterial segments within each genotype. Because data distributions did not meet normality assumptions, comparisons were conducted using the Mann–Whitney *U* test. For collagen fibers, data are presented as median. For elastin fibers, percentile P25 was used to specifically assess the lower tail of the distribution, reflecting the subpopulation of thinner fibers, as the rest of the population was similar between groups. Percentile‐based comparisons were performed using the same statistical framework applied to median values. All the reported number of experiments represent biological replicates. All graphs show *p* value as: *p < 0.05; **p < 0.01; ***p < 0.001; ****p < 0.0001.

## 3 RESULTS

### 3.1 Effects of partial carotid ligation (PCL) on arterial flow dynamics

To document the effects of partial carotid artery ligation in causing disturbed flow, we analyzed flow velocity during systole and end‐diastole by ultrasonography (Figure 1b,c). Blood flow in LCA showed the expected marked flow reduction after PCL, both of systolic and end‐diastolic flow velocities, indicating patterns consistent with bidirectional flow. This scenario is similar to that originally described by Nam et al. (2009) and indeed entails the occurrence of oscillatory shear stress, a pathological flow pattern, mainly at the base of the ligated carotid artery (i.e., close to aorta). This reduction was consistently observed across all mice, with no differences between WT and TgPDIA1 groups, indicating that PDIA1 overexpression does not alter the hemodynamic impact of PCL (Figure 1d–h). The right carotid artery had mild compensatory increases in blood flow and was therefore used as an internal control. Given the accentuated disturbance in flow patterns in the proximal (vs. distal) ligated carotid artery, we systematically performed all subsequent histomorphometric analyses in separated portions denominated 1 (distal) and 2 (proximal, close to aorta) of each artery (Figure 1a). Both WT and TgPDIA1 mice showed, in the LCAs, increased total caliber, medial, neointimal and lumen areas 14 days after PCL, characterizing expansive (outward) remodeling in this model, with distinct patterns described as follows.

### 3.2 PDIA1 overexpression exacerbates outward vascular remodeling after partial carotid ligation

To investigate the impact of PDIA1 overexpression on vascular remodeling, carotid arteries were harvested at 2 and 14 days after PCL with emphasis on the 14‐day time point, when structural remodeling is fully evident. At 2 days post‐ligation (Figure S1a), TgPDIA1 mice already exhibited an increase in total vessel area compared with WT mice, indicative of mild early outward expansion (Figure S1b–d). This effect was more clearly observed in the more distal LCA1 segment, whereas the overall caliber of segment LCA2 and lumen area (Figure S1e–g) of both segments did not reach statistical significance. Medial area and wall area were also slightly increased in segment 1 from TgPDIA1 mice at this early time point (Figure S2a–f). No differences were detected in the wall tension index (see below) between genotypes (Figure S2g–i).

**FIGURE 2:**
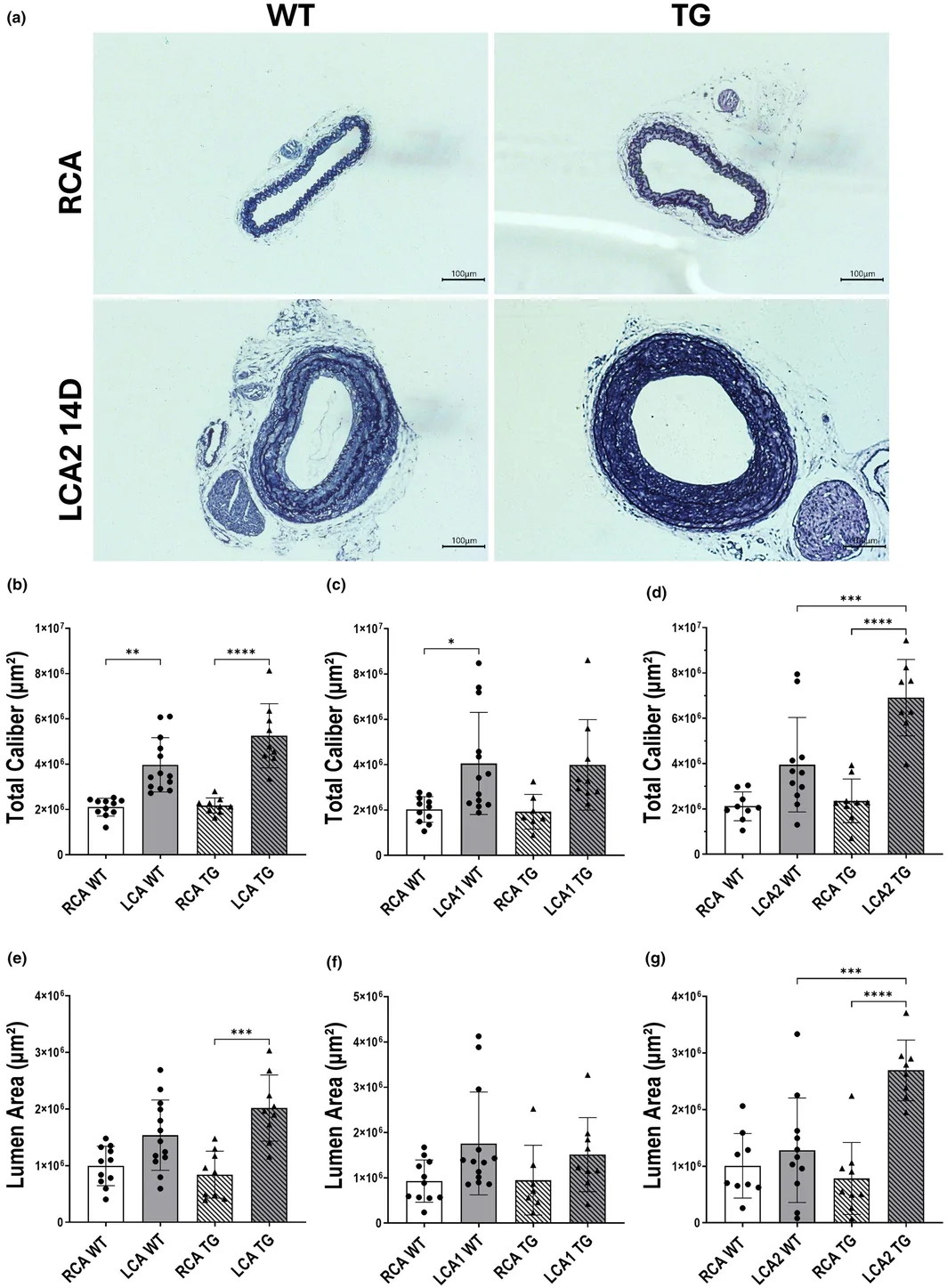
Histomorphometric analysis depicting exacerbated expansive remodeling in PDIA1‐overexpressing mice following PCL. (a) Verhoeff Van Gieson staining of right (RCA‐pooled) and left carotid artery segments (LCA‐pooled) from wild‐type (WT) or TgPDIA1 (TG) mice 14 days after PCL; (b–d) Total LCA caliber in WT and TG mice 14 days after PCL. The analysis was performed for pooled (LCA1 + 2) segments (b), or for LCA1 (c) or LCA2 (d) separately; (e–g) Similar to panels b–d, with analysis of lumen area. Each dot represents the RCA or LCA artery segment from one mouse (average of technical replicates) and reflects biological replicates. Magnification, 10×; Scale bar, 100 μm. Data are mean ± SD. *p < 0.05; **p < 0.01; ***p < 0.001; ****p < 0.0001. WT RCA1 *N* = 11; WT RCA2 *N* = 9; WT LCA1 *N* = 13; WT LCA2 *N* = 11; TG RCA1 *N* = 7; TG RCA2 *N* = 9; TG LCA1 *N* = 9; TG LCA2 *N* = 9.

**FIGURE 3:**
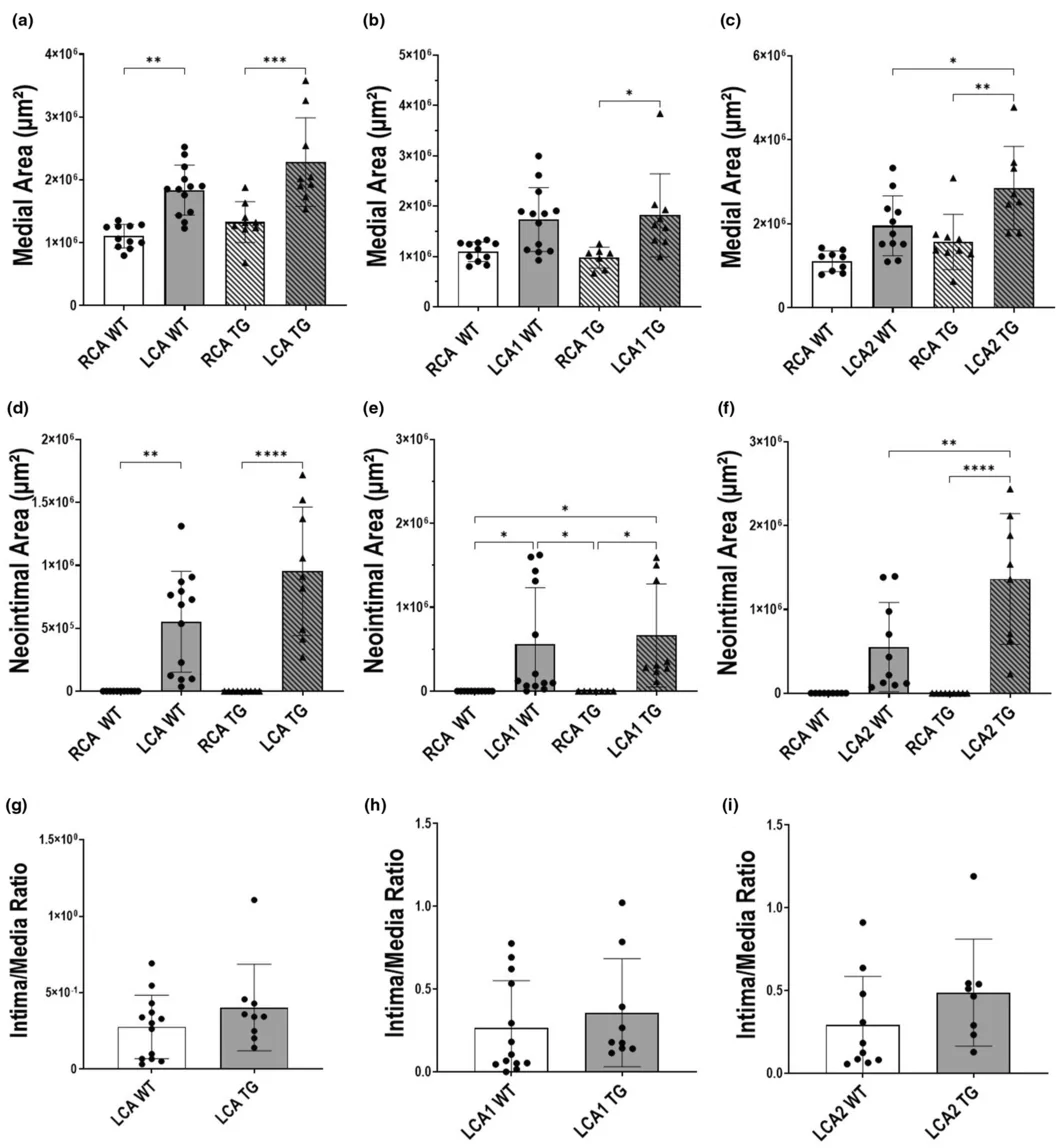
Enhanced medial and neointimal expansion in TgPDIA1 mice. Bar graphs depict quantifications of (a–c) Medial area and (d–f) Neointimal area, in both cases 14 days after PCL. Abbreviations and symbols follow those described in Figure 2 legend; (h, i) Intima‐to‐media area ratios 14 days after PCL for pooled LCA segments (g) or separately for LCA1 (h) or LCA2 (i) segments. While neointimal and medial areas were exacerbated in TgPDIA1 mice, the intima‐to‐media ratio was not significantly affected. WT RCA1 *N* = 11; WT RCA2 *N* = 9; WT LCA1 *N* = 13; WT LCA2 *N* = 11; TG RCA1 *N* = 7; TG RCA2 *N* = 9; TG LCA1 *N* = 9; TG LCA2 *N* = 9. *p < 0.05; **p < 0.01; ***p < 0.001; ****p < 0.0001.

**FIGURE 4:**
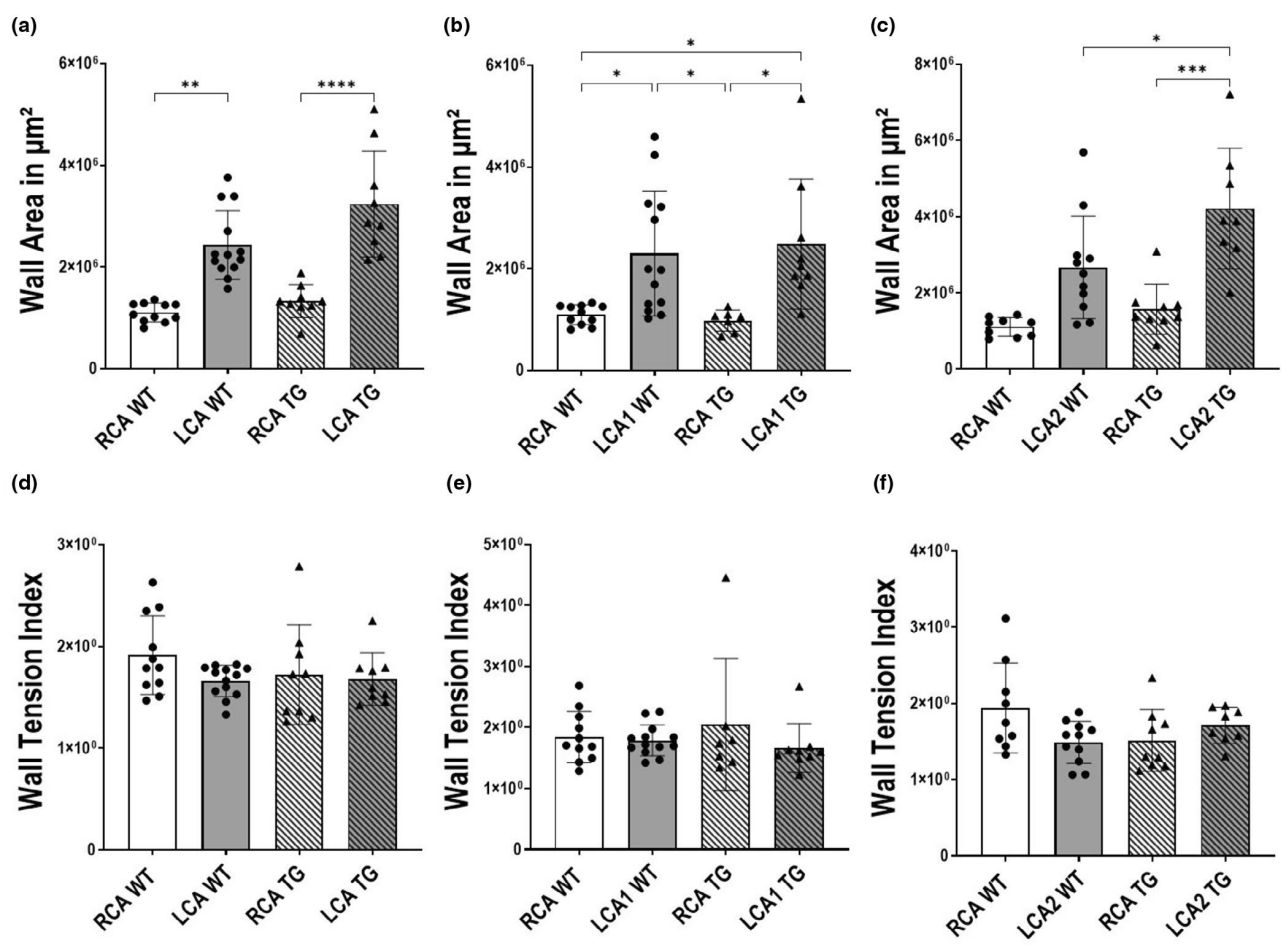
Wall area and wall tension index after PCL in WT and TG mice carotids. (a–c) Total wall area (media + intima) in pooled LCA segments and separately LCA1 (b) or LCA2 (c) segments; (d–f) Wall tension index (total vessel area normalized to wall area) shows no difference between WT and TG mice carotids, in RCA or pooled LCA segments (d) and separately for LCA1 (e) or LCA2 (f) segments. Abbreviations and symbols follow those described in Figure 2 legend. WT RCA1 *N* = 11; WT RCA2 *N* = 9; WT LCA1 *N* = 13; WT LCA2 *N* = 11; TG RCA1 *N* = 7; TG RCA2 *N* = 9; TG LCA1 *N* = 9; TG LCA2 *N* = 9. *p < 0.05; **p < 0.01; ***p < 0.001; ****p < 0.0001.

At 14 days after PCL, arterial remodeling was evident in both WT and TgPDIA1 mice carotids, but robustly exacerbated in the latter. This was shown by increase in total vessel caliber (area) (Figure 2a,d) in TgPDIA1 versus WT mice. Lumen area also followed the same behavior, with increase in TgPDIA1 versus WT mice (Figure 2e,g). In parallel, medial and neointimal areas were also significantly increased both in WT and TgPDIA1, but significantly enhanced in TgPDIA1 versus WT, particularly in segment 2 closer to aorta (Figure 3c,f). Meanwhile, intima/media area ratio was scarcely affected (Figure 3g–i). Accordingly, total wall area (media+intima) was also increased in TgPDIA1 versus WT mice (Figure 4c) in segment 2. Overall, such changes were less pronounced in segment 1, while the combined analysis of segments 1 + 2 also yielded an exacerbated expansive remodeling in the TgPDIA1 carotid versus WT (Figures 2, 3, 4). This indicates more pronounced vessel remodeling in the region with increasingly disturbed flow associated with oscillatory shear stress. Importantly, when total vessel area was normalized to wall area, an index used as a surrogate of vessel wall tension, no differences were observed between WT and TgPDIA1 mice (Figure 4d–f), suggesting that the enhanced outward remodeling in TgPDIA1 arteries follows a regulated process likely similar to those of WT, that is, able to reach a similar homeostatic tension setpoint despite distinct vessel calibers and wall mass. Consistent with this analysis, the normalization of results obtained in ligated arteries for their respective contralateral control arteries (LCA/RCA ratio) confirmed a similar significant increase in caliber and lumen area in TgPDIA1 mice, although the difference in medial area lost significance (Figure S3). Together, these data demonstrate that PDIA1 overexpression versus WT leads to an exacerbated outward vascular remodeling in response to disturbed flow with no evidence of disrupted global wall tension balance.

### 3.3 PDIA1 overexpression associates with reduced birefringent collagen despite increased total collagen content

**FIGURE 5:**
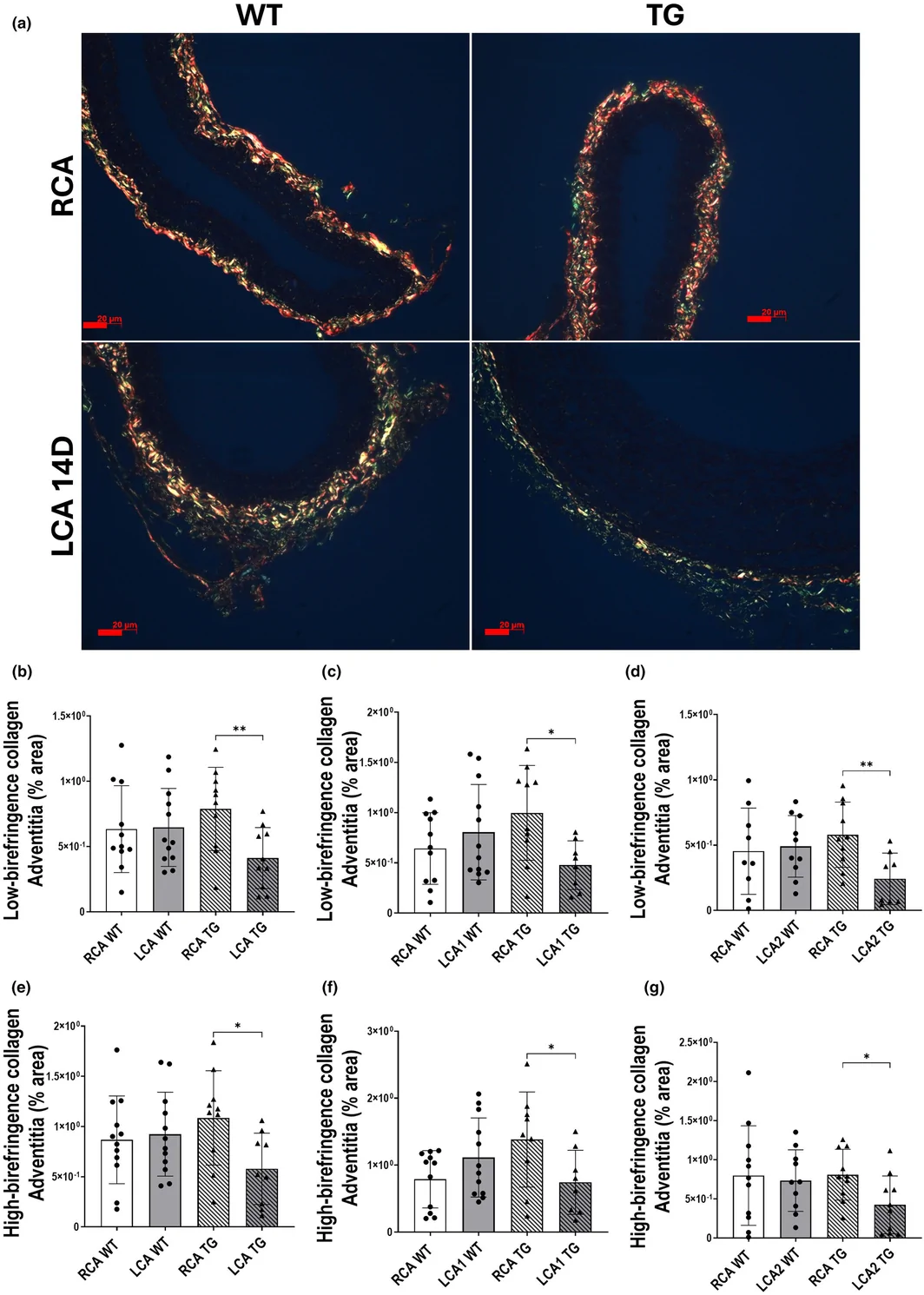
Analysis of birefringent collagen under polarized light microscopy in the adventitia of WT and TgPDIA1 carotid arteries after PCL. (a) Representative Picrosirius–stained sections under polarized light 14 days after PCL in WT and TG mice; (b–d) Quantification of low‐birefringent (green) collagen area fraction in the adventitia in RCA and pooled LCA segments (b) and separately for LCA1 (c) or LCA2 (d) segments; (e–g) Quantification of highly‐birefringent (red) collagen area fraction in the adventitia in RCA and pooled LCA segments (e) and separately for LCA1 (f) or LCA2 (g) segments (other corresponding measurements can be found in Figure S4). Abbreviations and symbols follow those described in Figure 2 legend. Magnification, 40×; Scale bar, 20 μm. WT RCA1 *N* = 12; WT RCA2 *N* = 11; WT LCA1 *N* = 12; WT LCA2 *N* = 10; TG RCA1 *N* = 10; TG RCA2 *N* = 10; TG LCA1 *N* = 9; TG LCA2 *N* = 9. *p < 0.05; **p < 0.01.

Analysis of ECM amount and architecture is essential to characterize vascular remodeling. Thus, we next evaluated collagen organization. Initially, we assessed collagen fiber organization using Picrosirius staining under polarized light (Junqueira et al., 1979) (Figure 5a). With this technique, collagen fibers show their birefringent properties, revealing a spectrum of color characterization from green (low birefringence) to yellow and red (high birefringence). The color spectrum varies according to fiber packing: green means loosely packed fibers and red means densely packed fibers (López De Padilla et al., 2021; Rich & Whittaker, 2005; Spiesz et al., 2011).

Ligated LCAs from TgPDIA1 mice exhibited significant (ca. 40%–60%) reduction in birefringent collagen compared with WT mice in the adventitial layer (Figure 5b–g). Both low‐birefringent (green) (5B‐D) and highly‐birefringent (red) (Figure 5e–g) collagen area fraction were significantly reduced in TgPDIA1 mice in both LCA segments 1 and 2, slightly accentuated for low‐birefringent collagen in segment 2 (Figure 5d). In contrast, collagen birefringence within the medial layer showed relatively lower values in general, with only minor detectable differences between TgPDIA1 and WT (Figure S4). These findings indicate that TgPDIA1 displays impaired fibrillar collagen organization detectable through these methods after PCL.

**FIGURE 6:**
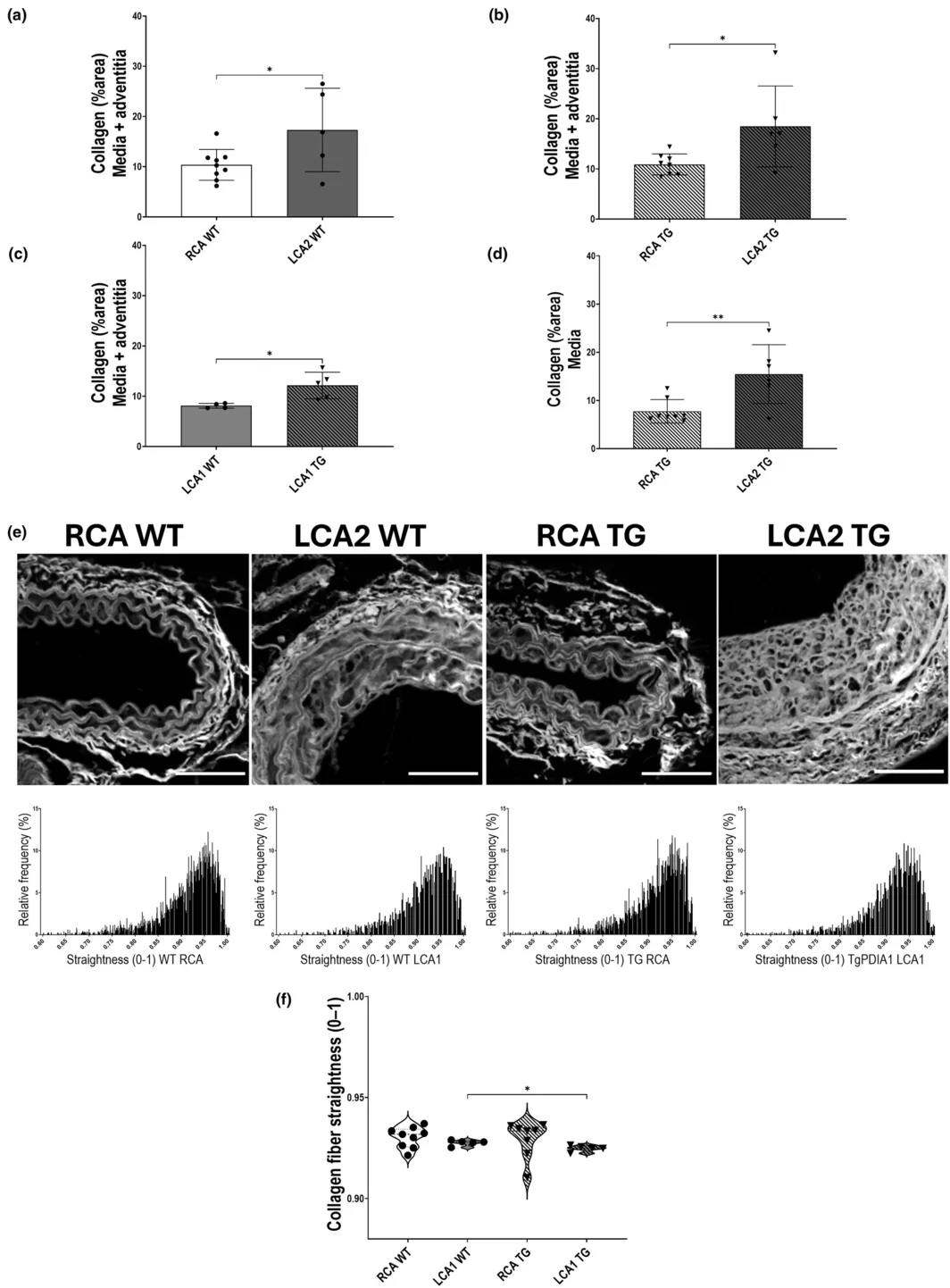
Analysis of collagen fiber architecture in WT and TgPDIA1 carotid arteries after PCL. Quantification of total collagen area (Picrosirius staining) demonstrating increased deposition in LCA2 (proximal) segment as a result of ECM remodeling after PCL, both in (a) WT and (b) TgPDIA1 carotids. Panels (c) and (d) indicate that collagen deposition in both LCA1 media + adventitia (c) and LCA2 media (d) was more pronounced in TG than in WT mice when compared with non‐ligated RCA; (e) Representative picture of confocal microscopy analysis of Picrosirius‐stained sections (upper panels) and frequency distribution of collagen fiber straightness (lower panels). (f) Increased population of curved fibers identified in LCA1 segments after PCL, depicting a slightly enhanced value for TG versus WT mice. Abbreviations and symbols follow those described in Figure 2 legend. Magnification, 40×; Scale bar, 50 μm. From (a–d): WT RCA1 *N* = 9; WT LCA1 *N* = 4; WT LCA2 *N* = 5; TG RCA1 *N* = 8; TG LCA1 *N* = 5; TG LCA2 *N* = 6. From (f): WT RCA1 *N* = 9; WT LCA1 *N* = 5; TG RCA1 *N* = 8; TG LCA1 *N* = 5. *p < 0.05; **p < 0.01.

In parallel, collagen was analyzed after Picrosirius staining by fluorescence confocal microscopy, which detects collagen fibers independently of birefringence (i.e., independently of their high‐level fibrillar organization) and therefore reflects total collagen content, including less organized and/or immature fibers (Lattouf et al., 2014) (Figure 6e). In contrast with the optical analysis, confocal analysis revealed an increase in collagen‐positive area in ligated carotid arteries from TgPDIA1 mice compared with WT (Figure 6c). This apparent discrepancy indicates that although total collagen content is increased in TgPDIA1 arteries, a substantial fraction fails to acquire the supramolecular organization required to generate strong birefringence. Thus, PDIA1 overexpression exacerbates collagen accumulation accompanied by its impaired maturation and/or organization during flow‐induced vascular remodeling.

### 3.4 PDIA1 overexpression associated with changes in collagen and elastin fiber architecture

To gain further insight into how PDIA1 overexpression affects overall ECM architecture, we used the CT‐FIRE software to analyze individual collagen or elastic fibers. Among the morphometric parameters extracted from images similar to those shown in Figure 6e for collagen and Figure 2a for elastin, some parameters were discriminative between genotypes. In RCA, TgPDIA1 mice exhibited only subtle differences in collagen fiber straightness compared with WT mice. However, at 14 days after PCL, TgPDIA1 ligated arteries presented an increased population of curved fibers (close to 0) compared to WT mice (Figure 6e,f). These changes are consistent with a less organized collagen network in TgPDIA1 mice in the course of disturbed flow‐induced vascular remodeling.

**FIGURE 7:**
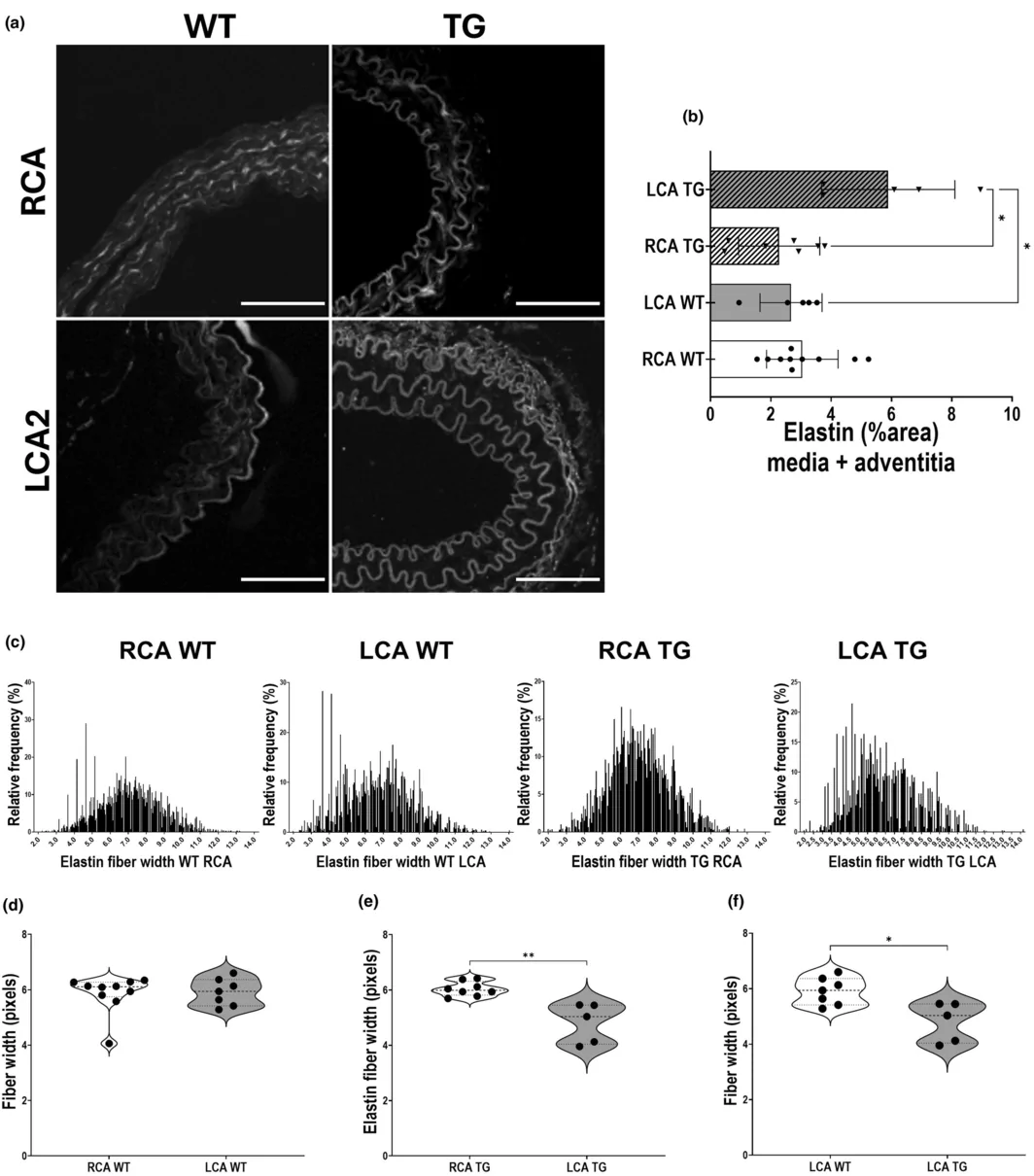
Analysis of elastin fiber profile in WT and TgPDIA1 carotid arteries after PCL. (a) Representative example of confocal microscopy image depicting elastin autofluorescence 14 days after PCL in WT and TG mice; (b) Quantification, linked to panel A, of elastin area in RCA and LCA segments depicting an exacerbated signal in the TG LCA after PCL; (c) Corresponding distribution of elastin fiber width in RCA and LCA segments from WT and TG mice carotids 14 days after PCL; (d–f) Violin‐plot graphs depicting analysis of elastic fiber width in RCA and LCA segments from WT and TG mice carotids 14 days after PCL. WT mice maintain LCA fiber width profile after PCL (d), in contrast to TG carotids, which exhibit thinner fibers compared either to TG RCAs (e) or WT LCA (f). Magnification, 40×; Scale bar, 50 μm. From (b) WT RCA1 *N* = 10; WT LCA1 *N* = 5; WT LCA2 *N* = 5; TG RCA1 *N* = 7; TG LCA1 *N* = 5; TG LCA2 *N* = 5. From (d–f) WT RCA1 *N* = 10; WT LCA1 *N* = 5; WT LCA2 *N* = 5; TG RCA1 *N* = 8; TG LCA1 *N* = 5. *p < 0.05; **p < 0.01.

In parallel, analysis of elastin fibers (Figure 7c–f) revealed that in ligated arteries from TgPDIA1 mice, elastin fibers were significantly thinner compared with WT, as reflected by a smaller population of wider fibers (Figure 7f). Also, this fiber population pattern in TgPDIA1 mice can be observed when comparing collagen fibers from RCA with LCA (Figure 7e). No changes were noted in WT mice (Figure 7d). Interestingly, elastin autofluorescence from confocal images (Figure 7a) shows increased elastin content in ligated TgPDIA1 carotid arteries (Figure 7a,b). These data indicate that PDIA1 overexpression also disrupts the structural organization of elastin networks during vascular remodeling.

### 3.5 Immunohistochemistry analysis of the vessel wall

**FIGURE 8:**
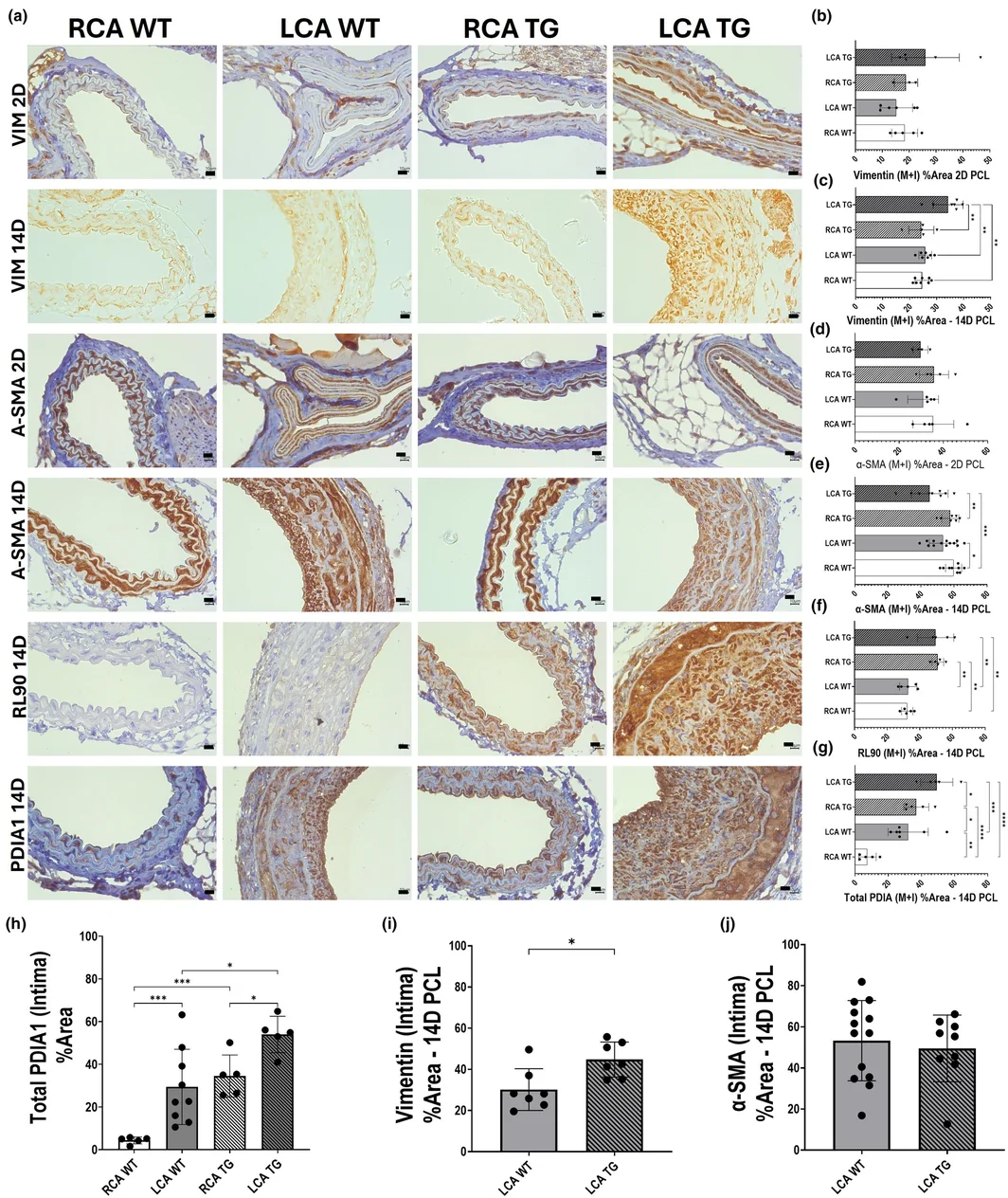
Immunohistochemical analysis of RCA and LCA segments from WT and TG mice carotids 14 days after PCL. (a) From top to bottom: Immunostaining of vimentin 2 or 14 days after PCL, alpha‐actin 2 or 14 days after PCL, PDIA1 with RL90 mouse antibody (which reacts well with the transgenic rat PDIA1 but poorly with endogenous mouse PDIA1) and PDIA1 with the PDIA1 N1N3 rabbit antibody (which reacts with both endogenous and transgenic PDIA1); the PDI stainings were14 days after PCL; (b–g) Quantitative analysis corresponding to the respective left panels in Figure 8a. Each dot corresponds to RCA or LCA from one mouse. The analyses included all the vessel layers; (h–k) Quantitative analysis focused on protein expressions at the innermost intimal layer (1.5 μm from lumen) 14 days after PCL in LCA. Data are shown from PDIA1 in RCA from WT versus TG mice (h), PDIA1 in LCA from WT versus TG mice (i), vimentin in LCA from WT versus TG mice (j) and alpha‐actin in LCA from WT versus TG mice. Abbreviations and symbols follow those described in Figure 2 legend. Magnification, 40×; Scale bar, 10 μm. Group number is described in Table S1. *p < 0.05; **p < 0.01; ***p < 0.001; ****p < 0.0001.

We next addressed the effects of PDIA1 overexpression on some phenotypic characteristics of the vessel wall in the absence or not of PCL through immunohistochemistry (Figure 8). After fourteen days, TgPDIA1 ligated arteries exhibited a significant increase in the expression of vimentin, while there were no changes in RCAs (Figure 8a,b), indicating remodeling of the intermediate filament network. Expression of alpha‐actin, which reflects a contractile VSMC phenotype, was unaltered in the RCA of TgPDIA1 mice, in agreement with our previous data (Fernandes et al., 2020). In the pooled LCA segment, alpha‐actin expression was slightly reduced in WT mice after 14 days of PCL and this reduction was exacerbated in TgPDIA1 mice (Figure 8a,e). Cell density in the vessel wall was significantly decreased in TgPDIA1 arteries at 2 days after PCL, possibly due to increased cell death. In contrast, at 14 days after PCL cell density was significantly increased in TgPDIA1 ligated arteries (Figure S5), consistent with a partial loss of VSMC contractile phenotype. Similar results were observed in the innermost intimal layer of cells (1.5 μm distance from lumen), occupied by endothelial and putatively other cells more directly exposed to the effects of disturbed flow. In this layer, TgPDIA1 ligated arteries exhibited increased expression of vimentin (Figure 8i) and a tendency to decreased expression of alpha‐actin (Figure 8j) compared with WT mice. This indicates that endothelial cells are also likely involved in the phenotypic reprogramming of the vessel wall, as indeed shown recently for this model (Park et al., 2025) and this effect seems stimulated in TgPDIA1 mice (Figure 8h,i). In TgPDIA1 mice, detection of the PDIA1 transgene via the RL90 antibody (which preferentially reacts with the rat PDI transgene rather than endogenous PDIA1) revealed a distinct spatial distribution (Figure 8f), with relatively stronger staining at the endothelial/peri‐luminal region (Figure 8a), in contrast to the more diffuse distribution of total PDIA1 detectable by another antibody reactive to mouse as well as rat PDI (Figure 8a).

### 3.6 Exacerbated expansive remodeling in TgPDIA1 mice following total carotid artery occlusion

**FIGURE 9:**
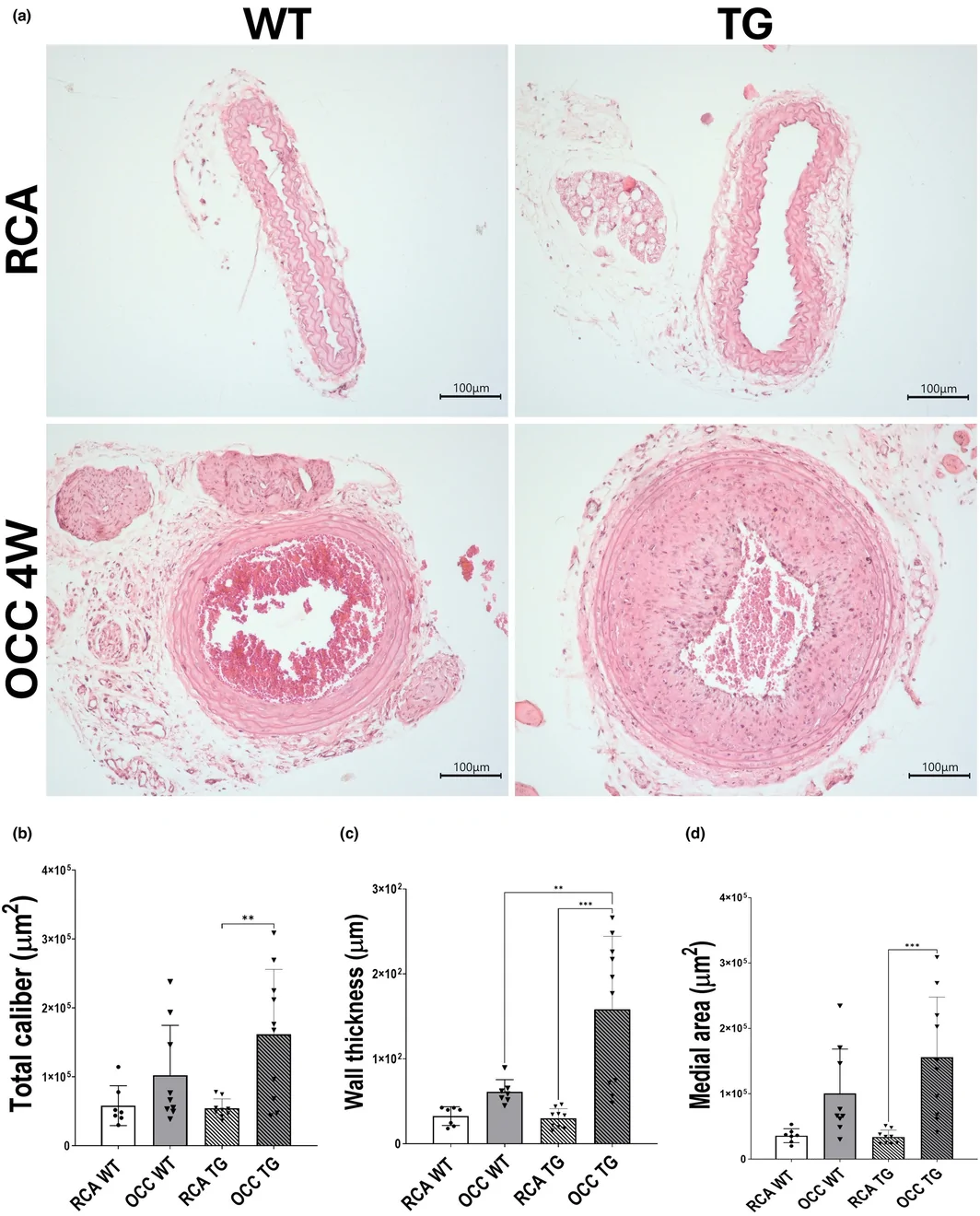
Histomorphometric analysis depicting exacerbated expansive remodeling in PDIA1‐overexpressing mice 28 days after total carotid artery ligation. (a) Verhoeff Van Gieson staining of control (RCA) and occluded (OCC) arteries 14 days after LCA ligation in WT and TG mice; (b–d) Quantitative analysis of total caliber (b), wall thickness (c) and medial area (d) in RCA and OCC carotids from WT and TG mice. Symbols are similar to those described in Figure 2 legend. Magnification, 10×; Scale bar, 100 μm. WT RCA *N* = 7; WT OCC *N* = 9; TG RCA *N* = 9; TG OCC *N* = 10. **p < 0.01; ***p < 0.001.

To understand whether PDIA1 overexpression would also promote expansive vascular remodeling in another model involving disturbed blood flow, we explored the model of total carotid artery occlusion. In this case, the disturbed blood flow pattern consists of low shear stress and the vascular response is likely to involve an additional burden of platelet activation/thrombosis in addition to inflammation (Kumar & Lindner, 1997), as indeed observed in our histological samples (Figure 9a). Our data indicated that despite enhanced variability of vascular responses 28 days after total carotid occlusion versus the partial occlusion model, TgPDIA1 mice also exhibited enhanced total vessel caliber, wall thickness, and medial area, consistent with exacerbated arterial remodeling (Figure 9b–d). Lumen area was difficult to quantify precisely given the high prevalence of mural thrombosis, so it was excluded from analysis.

## 4 DISCUSSION

Our results show that arteries from mice with global constitutive overexpression of PDIA1 undergo accentuated expansive vascular remodeling in the PCL model, with increased total vessel diameter, lumen caliber, medial, and neointimal areas. Such effects were more pronounced in the proximal carotid segment closer to the aorta, indicating a synergistic interaction between PDIA1 overexpression and the extent of flow disturbances, since oscillatory shear stress is more pronounced at this location. The effects of PDIA1 overexpression associate with lower organization of collagen fibrils and enhanced neointima formation. Meanwhile, such enhanced flow‐dependent remodeling appears to keep regulatory homeostasis, since the ratio between overall caliber and wall area was unaltered versus WT mice. Analogous effects of transgenic PDIA1 overexpression also occurred in the model of total carotid artery occlusion. These results are in line with our previous observations that enhanced PDIA1 expression supports expansive vessel remodeling in a model of vascular injury (Tanaka et al., 2016). A similar association between PDIA1 and outward remodeling is also observed in atherosclerotic human arteries (Muller et al., 2013; Tanaka et al., 2016). Thus, the present data add further evidence that overexpression of PDIA1 per se, whether forced or associated with endogenous signaling programs, is sufficient to promote an expanded vessel structure during remodeling, which sustains or even increases vascular lumen.

A relevant question regarding PDIA1 effects is the relative role of extracellular (peri/epicellular) versus total (extra + intracellular) pools. Our previous evidence indicates roles of peri/epicellular PDIA1 in supporting expansive remodeling during the late phase of vascular repair after injury (Tanaka et al., 2016). Moreover, such extracellular PDIA1 pool accounts for mechanoadaptive actin cytoskeleton remodeling, lower noise of force distribution and localized RhoGTPase activation (Pescatore et al., 2012), as well as redox modulation of integrins (Essex, 2004; Furie & Flaumenhaft, 2014). Immuno‐neutralization of extracellular PDIA1 abrogates PDGF‐dependent VSMC migration persistence with preserved total distance and velocity, while total PDIA1 silencing totally prevents directional VSMC migration (Tanaka et al., 2019), a key mechanism of neointima formation. In parallel, intracellular PDIA1 associates with Nox NADPH oxidase regulation (Janiszewski et al., 2005), NO availability (Leite et al., 2003), endothelial migration (Nagarkoti et al., 2023) and regulation of ER stress (Eletto et al., 2014). Therefore, it is likely that both intra and extracellular PDIA1 concur to regulate vessel remodeling.

Another question is: PDIA1 from which cell contributes to exacerbate vascular remodeling? Vessel remodeling is a complex integrative event involving all vascular wall cell types and their interaction with ECM (Tanaka & Laurindo, 2017; Zhang et al., 2025). Both endothelial and medial layers depicted enhanced PDIA1 expression in the present study, corroborating our previous observations in this model (Tanaka et al., 2024). Previous evidence indicates that PCL promotes endothelial expression of several mechanosensitive genes (Ni et al., 2010), but in particular of inflammatory genes (Ni et al., 2011), able to trigger partial reprogramming into other phenotypes, including mesenchymal and even immune cell‐like transitions (Park et al., 2025). Therefore, this model entails mechanically triggered inflammatory vascular adaptations (Tarbell et al., 2014), with secondary changes in ECM (Tamargo et al., 2023). Since the primary stimulus for vessel remodeling is low/oscillatory shear stress, which is sensed mainly by endothelial cells (Andueza et al., 2020; Nam et al., 2009), it is plausible that endothelial cell PDIA1 overexpression is particularly crucial for VSMC phenotypic adaptations (Tamargo et al., 2023) and exacerbated vascular remodeling. In parallel, overexpression of VSMC PDIA1 observed in the present study also occurs during vascular injury repair (Tanaka et al., 2016, 2024) and seems directly involved in vessel remodeling, since we showed previously that a perivascular gel containing neutralizing antibody against PDIA1 promoted loss of total caliber without changes in neointima (Tanaka et al., 2016). Furthermore, platelet PDIA1 contributes to their activation in the early stages of vascular response (Tanaka & Laurindo, 2017), amplifying thromboinflammation (Flaumenhaft & Furie, 2016). Finally, inflammatory cells such as macrophages and neutrophils also robustly express PDIA1 (de A. Paes et al., 2011; Santos et al., 2009) and its overexpression in these cells may also amplify inflammation and vessel remodeling (Tanaka et al., 2016). Thus, it is likely that the overexpressed PDIA1 from multiple cell types plays converging roles to determine exacerbated vessel response. Future studies with cell‐specific PDIA1 deletion may contribute to discern roles of each cell type.

Our evidence for a maintained setpoint of the vessel response to disturbed flow, as suggested by conserved ratios of total vessel area to wall area, suggests that the exacerbated remodeling induced by PDIA1 overexpression keeps mechano‐sensitive homeostasis during vessel expansion, rather than being an unregulated proinflammatory event. This accords with the hypothesis that PDIA1 acts as an upstream regulator of mechanoadaption, as suggested from our previous studies (Tanaka et al., 2016, 2019, 2024). The precise sensor mechanisms accounting for this putative servomechanism remain to be established, but likely involve the VSMC‐ECM binomium (Heusch et al., 2014). Our TgPDIA1 mice exhibited a decreased amount of organized collagen fibrils after PCL, despite an enhanced amount of less organized fibers, which exhibited enhanced curvature versus WT mice. This further suggests that PDIA1 overexpression associates with impaired collagen organization despite increased amount, in line with previous evidence that PDIA1 expression governs collagen architecture (Van Varik et al., 2012). We propose a model in which exacerbated collagen cross‐links during vessel remodeling in TgPDIA1 mice tend to promote an enhanced vascular tension, composing increased circumferential, radial and longitudinal wall stresses. This tendency, however, is counteracted by enhanced wall thickening organized outwardly, which normalizes wall stress at the expense of an enlarged overall vessel caliber.

In line with prior studies using lineage tracing and single cell genomics in PCL model, endothelial cells seemingly undergo substantial phenotypic reprogramming and even without lipid overload may transition to mesenchymal, VSMC, immune and foam cell phenotypes (Park et al., 2025), which contribute to neointima formation (Tamargo et al., 2023). In our study, endothelial PDIA1 overexpression may have contributed to transitions towards phenotypes composing the exacerbated neointimal and even medial layers. PDIA1 loss of function induces endothelial cell senescence via Drp‐mediated mitochondrial fission (Kim et al., 2018) and impairs VEGFR‐dependent migration (Nagarkoti et al., 2023). Disturbed flow in PCL upregulates PDIA1 via microRNA‐204, directly connecting hemodynamic stress to PDIA1 expression in intima and media/adventitia (Tanaka et al., 2024). An additional aspect of PDIA1 overexpression is its association with phenotypic VMSC changes, both in isolated cells and transgenic mice (Fernandes et al., 2020). This was also shown after microRNA modulation (Tanaka et al., 2024) and PDIA1 loss of function during vessel repair (Tanaka et al., 2016). Whether and how VSMC phenotype associates with enhanced remodeling in our model deserves further study, but might relate to recapitulation of mesenchymal‐like phenotypes (Yap et al., 2021), as we showed for aortic VSMC from Marfan Syndrome mice (Nolasco et al., 2020). Future studies using lineage tracing and single cell transcriptomics should contribute to understanding such mechanisms.

In addition to ECM organization and cell phenotype modulation, reorganization of the actin cytoskeleton, particularly in VSMC, is also an important remodeling mechanism, as documented previously during vascular repair (Wesselman & De Mey, 2002). Importantly, the roles of extracellular PDIA1 in fine‐tuning of the actin cytoskeleton in VSMC, accompanied by evidence for beta1‐integrin oxidation (Tanaka et al., 2019), were previously shown (Tanaka & Laurindo, 2017). Additionally, extracellular PDIA1‐dependent mechanoadaptive effects in isolated associate with actin cytoskeleton reorganization (Tanaka et al., 2016). The increased vimentin expression in TgPDIA1 mice after PCL was notable in our study. Vimentin is reportedly required for flow‐dependent remodeling (Lund et al., 2010), associates with β1‐integrin assembly in fibroblasts (Ostrowska‐Podhorodecka et al., 2021), supports leukocyte adhesion during inflammation (Nieminen et al., 2006) and stabilizes collagen α1 and α‐2 mRNA in fibrosis (Challa & Stefanovic, 2011). Vimentin KO mice exhibit less stiff arteries during vessel remodeling via Notch signaling (Langlois et al., 2017; van Engeland et al., 2019). This is consistent with intermediate filaments also playing roles in PDIA1‐supported vessel remodeling.

Our work has several limitations. First, cellular and molecular mechanisms involved in PDIA1‐dependent exacerbated remodeling remain unclear. Also, forced PDIA1 overexpression may not fully reproduce all aspects of the overexpression occurring in physiological remodeling in vivo regarding, for example, distinct post‐translational modifications and subcellular locations. The FVB phenotype of our mice may influence the extent and type of vascular responses. For example, low flow‐induced vessel remodeling and neointima formation are exacerbated in FVB background versus other strains, including C57BL‐6 (Harmon et al., 2000; Inoue et al., 2007; Östergren et al., 2015). On the other hand, aortic dissection promoted by lysyl oxidase inhibition is much less evident in FVB mice (Franklin et al., 2024). Thus, future work in other genetic backgrounds may reveal some distinct aspects of PDI‐dependent vessel remodeling. However, we should note that prior studies in rabbits also depicted anti‐constrictive remodeling effects of PDIA1 (Tanaka et al., 2016). Regarding assessment of the collagen ECM, small variations in tissue orientation may alter angle measurements independently of actual changes in collagen organization, a reason why we could not analyze fiber angle by CT‐FIRE software.

Overall, our data, together with previous results, offer further robust evidence that PDIA1 overexpression per se acts as a novel (patho)physiologically relevant upstream mediator of expansive/anti‐constrictive vascular remodeling, a fundamental disease mechanism. On an extended perspective, PDIA1 and possibly other components of the ER redoxome, particularly of the so‐called ER‐dependent outreach redoxome (ERDOR) (Oliveira et al., 2026), emerge as relevant orchestrators of the direction and magnitude of vascular remodeling, likely via redox mechanisms (Tanaka & Laurindo, 2017) governing concerted transitions involving intercellular communication, cellular phenotype, mechanotransduction, and ECM architecture.

## AUTHOR CONTRIBUTIONS

**Júlia Martins Felipe de Souza:** Conceptualization; data curation; formal analysis; investigation; methodology; validation; visualization. **Tiphany Coralie De Bessa:** Data curation; formal analysis; validation; visualization. **Amanda de Almeida Silva:** Methodology; software. **Carolina Gonçalves Fernandes:** Conceptualization; formal analysis; investigation; methodology; validation. **Leonardo Yuji Tanaka:** Conceptualization; methodology; supervision. **Francisco Rafael Martins Laurindo:** Conceptualization; data curation; funding acquisition; project administration; resources; supervision; validation.

## FUNDING INFORMATION

The authors are members of the CEPID Redoxoma Network, supported by Fundação de Amparo à Pesquisa do Estado de São Paulo (FAPESP) (Grant 2013/07937‐8). This work was also supported by Fundação Zerbini, FAPESP grant 2024/16069‐4 and scholarship grants: 2021/14131‐6 (to JMFS); 2018/07511‐4 and 2024/02534‐7 (to TCB); LYT was supported by FAPESP Young Investigator Grant 2018/07230‐5. CGF was supported by CAPES scholarship 88882.315650/2019‐01.

## CONFLICT OF INTEREST STATEMENT

The authors declare no conflicts of interest.

## Supporting information


**Figure S1.** Histomorphometric analysis of RCA or LCA remodeling 2 days after LCA PCL in WT or TG mice. (a): Representative examples of arterial sections stained by Verhoeff Van Gieson method 2 days after PCL; (b–d) Quantitative morphometric analysis depicting total vascular caliber in RCA and pooled LCA segments (b) and separately for LCA1 (c) or LCA2 (d) segments; (e–g): Similar to panels b–d, depicting arterial lumen area. Abbreviations and symbols follow those described in Figure 2 legend. Each dot represents the RCA or LCA artery segment from one mouse (average of technical replicates) and reflects biological replicates. Magnification,10×; Scale bar,100 μm. WT RCA1 *N* = 5; WT RCA2 *N* = 5; WT LCA1 *N* = 4; WT LCA2 *N* = 3; TG RCA1 *N* = 5; TG RCA2 *N* = 5; TG LCA1 *N* = 5; TG LCA2 *N* = 5.
**Figure S2.** Histomorphometric analysis (continued) of RCA or LCA remodeling 2 days after LCA PCL in WT or TG mice. (a–c): Quantitative morphometric analysis depicting total vascular caliber in RCA and pooled LCA segments (a) and separately for LCA1 (b) or LCA2 (c) segments; (d–f): Similar to panels a–c, depicting vascular wall area; (g–i): Similar to panels a–c, depicting wall tension index. Abbreviations and symbols follow those described in Figure 2 legend. WT RCA1 *N* = 5; WT RCA2 *N* = 5; WT LCA1 *N* = 4; WT LCA2 *N* = 3; TG RCA1 *N* = 5; TG RCA2 *N* = 5; TG LCA1 *N* = 5; TG LCA2 *N* = 5.
**Figure S3.** Histomorphometric analysis comparing RCA versus corresponding LCA areas 14 days after PCL in WT or TG mice. (a–c): Quantitative morphometric analysis depicting total vascular caliber (measured as area) in RCA versus pooled LCA segments (a) and separately for LCA1 (b) or LCA2 (c) segments; (d–f): Similar to panels a–c, depicting analysis of medial areas; (g–i): Similar to panels a–c, depicting lumen areas. Abbreviations and symbols follow those described in Figure 2 legend. WT RCA1 *N* = 11; WT RCA2 *N* = 9; WT LCA1 *N* = 13; WT LCA2 *N* = 11; TG RCA1 *N* = 7; TG RCA2 *N* = 9; TG LCA1 *N* = 9; TG LCA2 *N* = 9.
**Figure S4.** Analysis of birefringent collagen under polarized light microscopy in the medial layer of WT and TgPDIA1 carotid arteries after PCL. (a–c): Quantification of low‐birefringent (green) collagen area fraction in the medial layer in RCA and pooled LCA segments (a) and separately for LCA1 (b) or LCA2 (c) segments; (d–f): Quantification of highly‐birefringent (red) collagen area fraction in the medial layer in RCA and pooled LCA segments (d) and separately for LCA1 (e) or LCA2 (f) segments. Abbreviations and symbols follow those described in Figure 2 legend. WT RCA1 *N* = 12; WT RCA2 *N* = 11; WT LCA1 *N* = 12; WT LCA2 *N* = 10; TG RCA1 *N* = 10; TG RCA2 *N* = 10; TG LCA1 *N* = 9; TG LCA2 *N* = 9.
**Figure S5.** Morphometric analysis of cell density in carotid segments from WT and TG arteries 14 (panels a–c) or 2 (panels d–f) days after LCA PCL. (a): Representative examples of arterial sections harvested 14 days after PCL and stained by hematoxylin/eosin; graph depicts quantitative morphometric analysis of cell density in RCA and pooled LCA segments from WT or TG mice; (b–c): Cell density analysis separately for LCA1 (b) or LCA2 (c) segments; (d–f): Similar to panels A‐C, depicting results of the analysis 2 days after PCL. Abbreviations and symbols follow those described in Figure 2 legend. Magnification, 40×; Scale bar, 10 μm. From (a–c): WT RCA1 *N* = 13; WT RCA2 *N* = 13; WT LCA1 *N* = 13; WT LCA2 *N* = 10; TG RCA1 *N* = 9; TG RCA2 *N* = 9; TG LCA1 *N* = 11; TG LCA2 *N* = 9. From (d–f): WT RCA1 *N* = 4; WT RCA2 *N* = 4; WT LCA1 *N* = 4; WT LCA2 *N* = 5; TG RCA1 *N* = 5; TG RCA2 *N* = 5; TG LCA1 *N* = 5; TG LCA2 *N* = 5.
**Table. S1.** Number of samples used for each analysis. Groups for partial carotid ligation (a) after 2 days, (b) after 14 days and (c) total carotid ligation after 3 weeks.

## Data Availability

All data in this study are available from the corresponding author upon request.
