# Supplementary material for: Exacerbated expansive vascular remodeling due to disturbed flow in male mice overexpressing protein disulfide isomerase‐A1
Source: Physiol Rep. 2026 Aug 30;14(17):e71088. doi: 10.14814/phy2.71088 (PMC13527409; doi:10.14814/phy2.71088)
Supplement: Supplementary file 1 [file PHY2-14-e71088-s001.docx]

**Exacerbated expansive vascular remodeling due to disturbed flow in male mice overexpressing Protein Disulfide Isomerase-A1**

**(Supplementary Data)**

Júlia Martins Felipe de Souza ^1^

Tiphany Coralie De Bessa ^1^

Amanda de Almeida Silva ^2^

Carolina Gonçalves Fernandes ^3^

Leonardo Yuji Tanaka ^1, 4^

Francisco Rafael Martins Laurindo ^1^

From:

^1^ Vascular Biology Lab (LVascBio), LIM-64, Heart Institute, University of São Paulo School of Medicine (FMUSP), Sao Paulo, SP-Brazil;

^2^ Laboratory of Hypertension, Heart Institute (InCor), University of São Paulo School of Medicine, São Paulo, SP-Brazil;

^3^ Santa Casa de São Paulo, School of Medical Sciences (FCMSCSP) Sao Paulo, SP-Brazil;

^4^ Redox Mechanobiology Laboratory, Federal University of São Paulo (UNIFESP), School of Medicine, Sao Paulo, SP-Brazil.

**Corresponding author:**

Francisco R.M. Laurindo, MD PhD

Laboratorio de Biologia Vascular (LVascBio), LIM-64, Instituto do Coracao (InCor), Hospital das Clinicas HCFMUSP, Faculdade de Medicina, Universidade de São Paulo

Av. Eneas Carvalho Aguiar, 44, Annex 2, 9^th^ floor, CEP 05403-000 – São Paulo – Brazil.

*Phone*: +55 (11) 2661 5185

E-mail: francisco.laurindo@hc.fm.usp.br

**Supplementary Fig. 1: Histomorphometric analysis of RCA or LCA remodeling 2 days after LCA PCL in WT or TG mice.** (A): Representative examples of arterial sections stained by Verhoeff Van Gieson method 2 days after PCL; (B-D) Quantitative morphometric analysis depicting total vascular caliber in RCA and pooled LCA segments (B) and separately for LCA1 (C) or LCA2 (D) segments; (E-G): Similar to panels B-D, depicting arterial lumen area. Abbreviations and symbols follow those described in Fig.2 legend. Each dot represents the RCA or LCA artery segment fromone mouse (average of technical replicates) and reflects biological replicates. Magnification,10x; Scale bar,100µm. WT RCA1 N=5; WT A2 N=5; WT LCA1 N=4; WT LCA2 N=3; TG RCA1 N=5; TG RCA2 N=5; TG LCA1 N=5; TG LCA2 N=5.

**Supplementary Fig. 2: Histomorphometric analysis (continued) of RCA or LCA remodeling 2 days after LCA PCL in WT or TG mice.** (A-C): Quantitative morphometric analysis depicting total vascular caliber in RCA and pooled LCA segments (A) and separately for LCA1 (B) or LCA2 (C) segments; (D-F): Similar to panels A-C, depicting vascular wall area; (G-I): Similar to panels A-C, depicting wall tension index. Abbreviations and symbols follow those described in Fig.2 legend. WT RCA1 N=5; WT RCA2 N=5; WT LCA1 N=4; WT LCA2 N=3; TG RCA1 N=5; TG RCA2 N=5; TG LCA1 N=5; TG LCA2 N=5.

**Supplementary Fig. 3: Histomorphometric analysis comparing RCA vs. corresponding LCA areas 14 days after PCL in WT or TG mice***.* (A-C): Quantitative morphometric analysis depicting total vascular caliber (measured area) in RCA vs. pooled LCA segments (A) and separately for LCA1 (B) or LCA2 (C) segments; (D-F): Similar to panels A-C, depicting analysis of medial areas; (G-I): Similar to panels A-C, depicting lumen areas. Abbreviations and symbols follow those described in Fig.2 legend. WT RCA1 N=11; WT RCA2 N=9; WT LCA1 N=13; WT LCA2 N=11; TG RCA1 N=7; TG RCA2 N=9; TG LCA1 N=9; TG LCA2 N=9.

**Supplementary Fig 4: Analysis of birefringent collagen under polarized light microscopy in the medial layer of WT and TgPDIA1 carotid arteries after PCL.** (A-C): Quantification of low-birefringent (green) collagen area fraction in the medial layer in RCA and pooled LCA segments (A) and separately for LCA1 (B) or LCA2 (C) segments; (D-F): Quantification of highly-birefringent (red) collagen area fraction in the medial layer in RCA and pooled LCA segments (D) and separately for LCA1 (E) or LCA2 (F) segments. Abbreviations and symbols follow those described in Fig.2 legend. WT RCA1 N=12; WT RCA2 N=11; WT LCA1 N=12; WT LCA2 N=10; TG RCA1 N=10; TG RCA2 N=10; TG LCA1 N=9; TG LCA2 N=9.

**Supplementary Fig 5: Morphometric analysis of cell density in carotid segments from WT and TG arteries 14 (panels A-C) or 2 (panels D-F) days after LCA PCL.** (A): Representative examples of arterial sections harvested 2 days after PCL and stained by hematoxylin/eosin; graph depicts quantitative morphometric analysis of cell density in RCA and pooled LCA segments from WT or TG mice; (B-C): Cell density analysis separately for LCA1 (B) or LCA2 (C) segments; (D-F): Similar to panels A-C, depicting results of the analysis 14 days after PCL. Abbreviations and symbols follow those described in Fig.2 legend. Magnification,40x; Scale bar,10µm. From (A-C): WT RCA1 N=13; WT RCA2 N=13; WT LCA1 N=13; WT LCA2 N=10; TG RCA1 N=9; TG RCA2 N=9; TG LCA1 N=11; TG LCA2 N=9. From (D-F): WT RCA1 N=4; WT RCA2 N=4; WT LCA1 N=4; WT LCA2 N=5; TG RCA1 N=5; TG RCA2 N=5; TG LCA1 N=5; TG LCA2 N=5.

| (A) **2D PCL Analysis** | **WT RCA1** | **WT RCA2** | **WT LCA1** | **WT LCA2** | **TG RCA1** | **TG RCA2** | **TG LCA1** | **TG LCA2** |
| --- | --- | --- | --- | --- | --- | --- | --- | --- |
| *Morphometric Analysis* | 5 | 5 | 4 | 3 | 5 | 5 | 5 | 5 |
| *Cell Density* | 4 | 4 | 4 | 5 | 5 | 5 | 5 | 5 |
| *Immunohistochemistry: VIM 2D* | 5 | - | - | 6 | 3 | - | - | 5 |
| *Immunohistochemistry: SMA 2D* | 5 | - | - | 5 | 5 | - | - | 4 |

| (B) **14D PCL Analysis** | **WT RCA1** | **WT RCA2** | **WT LCA1** | **WT LCA2** | **TG RCA1** | **TG RCA2** | **TG LCA1** | **TG LCA2** |
| --- | --- | --- | --- | --- | --- | --- | --- | --- |
| *Doppler Ultrasound and Echocardiography Analysis* | 8 | - | 8 | - | 10 | - | 10 | - |
| *Morphometric Analysis* | 11 | 9 | 13 | 11 | 7 | 9 | 9 | 8 |
| *Cell Density* | 13 | 13 | 13 | 10 | 9 | 9 | 11 | 9 |
| *Collagen analysis under polarized light* | 12 | 11 | 12 | 10 | 10 | 10 | 9 | 9 |
| *Collagen analysis under confocal microscopy* | 9 | - | 4 | 5 | 8 | - | 5 | 6 |
| *Elastin analysis under confocal microscopy* | 10 | - | 5 | 5 | 7 | - | 5 | 5 |
| *Collagen fiber morphometry* | 9 | - | 5 | - | 8 | - | 5 | - |
| *Elastin fiber morphometry* | 10 | - | 7 | - | 8 | - | 5 | - |
| *Immunohistochemistry: VIM 14D* | 8 | - | - | 7 | 5 | - | - | 7 |
| *Immunohistochemistry: SMA 14D* | 9 | - | - | 13 | 7 | - | - | 9 |
| *Immunohistochemistry: RL90 14D* | 5 | - | - | 5 | 5 | - | - | 5 |
| *Immunohistochemistry: PDIA1 14D* | 5 | - | - | 7 | 5 | - | - | 5 |

| **(C) 3W OCC Analisis** | **WT RCA** | **WT OCC** | **TG RCA** | **TG OCC** |
| --- | --- | --- | --- | --- |
| *Morphometric Analysis* | 7 | 9 | 9 | 10 |

**Supplementary Table. 1: Number of samples used for each analisys.** Groups for partial carotid ligation (A) after 2 days, (B) after 14 days and (C) total carotid ligation after 3 weeks.
